# A Critical Inquiry into the Pathology of Scrofula; in Which the Origin of That Disease Is Accounted for on New Principles, and a New and Much Improved Method Is Recommended and Explained for the Treatment of It

**Published:** 1816-11

**Authors:** 


*A Critical Inquiry into the Pathology of Scrofula; in which the Origin of that Disease is accounted for on new Principles, and a new and much improved Method is recommended and explained for the Treatment of it.* By GEORGE HENNING, M.D. 8vo. pp. 256. London, 1815.

THE work of Dr. Henning has long been in our collection; and we have repeatedly but in vain, within the last year, attempted its regular analysis. There is something singularly unpromising and repulsive in the physiognomy of the book; and the external characters, in this instance, mark but too correctly the intellectual constitution. Dr. Henning evidently possesses erudition, industry, and talent. We regret to see them thus prostituted and abused. Children and idiots may be allowed to amuse themselves with blowing air-bubbles: The man of real sense and ability should be more honourably and usefully employed. Our review of the publication, although more desultory and concise than we are in the habit of instituting, will enable the reader to decide whether or not this reprehension be justly merited. Of the brevity of our notice, if practical value constitute, as it ought, the standard of mensuration, Dr. Henning will have no reason to complain.

The *Introduction* to the volume contains little worthy of remark. An assertion of Sydenham, respecting the practical advantages that would result from an accurate acquaintance with the natural history of diseases, seems wonderfully to have dazzled the Bristol physician, and forms, in fact, the polar star of his researches. But the correctness of this proposition may, without imputation of temerity, be questioned: for, if the proximate causes of a disease constitute an essential part of its natural history, we utterly despair of any real or decided progress being made in the study. The obscurity in which the nature and operation of these causes are, at present, enveloped, will probably for ever hide them from human research; and our English Hippocrates did well to qualify his assertion by a conditional. Archimedes would have moved the earth, *if* he could but have discovered a fulcrum for his lever.

The work consists of two Parts; the first comprehending researches in the natural history and pathology of scrofula; the latter, its chirurgical and medical treatment.

*Part the First.* In the *seven chapters* which form this division, Dr. Henning examines the etymology and import of the terms employed to designate the malady;* reviews

---

* χοιραδες is the Greek, and *scrofula* or *struma* the Latin term for the malady which we call *King's-evil.* The Greek noun is derived from *χοιρας, a pig*; and of this *scrofulæ* is a literal translation. Various reasons have been assigned for the application of this term to the disease. They all rest on some fancied resemblance of the latter to the pig, either in external shape, pathological character, or rapidity of propagation. Now χοιραδες denotes also *a rock rising above the sea;* and Dr. Henning is at wonderful pains to make it appear that scrofulous tumours more strongly resemble rocks than pigs: for "the human neck, when it is much studded with indurated glands, enlarged and elevated above the surface, really has a rocky or craggy appearance." Hence the Doctor supposes, that the text of Hippocrates has been corrupted in this, as in many other instances; and infers, that the "true reading is not χοιραδες, *scrofulæ,* but χοιραδωδες, *scopulosus*"—a supposition and inference certainly without any thing, except loose analogy and wanton imagination, to sanction and support them. Rev.

some of the theories invented to elucidate its nature:* then successively treats of the supposed hereditary and constitutional nature of scrofula; of its predisposing cause; of its proximate cause; of its seat; and lastly, of the analogy between scrofula and lues venerea. The two first chapters are scarcely deserving of any formal notice. In our examination of the remaining five, we shall concisely state the doctrines which they contain, and the reasonings or observations from which they are deduced; and, lastly, offer such remarks as our own views of the subject may suggest, and our own experience justify.

First then, scrofula is neither hereditary nor of constitutional origin. In proof of the former part of this assertion, it is argued, that were the disease, or predisposition to it, hereditary, it should be "constantly, not occasionally, inherited;" "all the children of the same bed" should alike become its victims. On the other hand, it is frequently seen in individuals not descended from scrofulous parents, and "the natives of the temperate climates, where scrofula is unknown, upon migrating to the cold and fluctuating regions of the north, are there invariably attacked by it." The second part of the position will be more fully illustrated as we proceed.

Secondly. The predisposing cause of scrofula is foreign to the body, and depends on peculiarities of climate.—Diseases of the superficial glands are endemial in countries, the atmosphere of which is generally damp and cold; while in warm and dry climates, and particularly within the tropics, they are utterly unknown. Bronchocele, which may be regarded as of a scrofulous nature, is very prevalent in the mountains of Switzerland, Genoa, and Piedmont, as in the most hilly situations of this country, but

* Peculiar acidity of the blood or chyle, viscidity of the lymph, and constitutional debility, are among the principal causes to which the developement of scrofula has been attributed. These theories, Dr. Henning very reasonably rejects. Whether he has erected on their ruins one less objectionable, will presently be seen. We cannot, however, refrain from smiling at the specimen of profound skill in experimental chemistry which the Doctor exhibits. He immerses slips of test-paper into the *recently drawn* blood of scrofulous patients, in order to discover whether or not any acidity be present. Now would not the addition of the colouring matter of the blood on the paper completely efface, or render obscure, any change of colour resulting from the presence in it of an uncombined acid? and might not such acid, if present, be discovered in the serum? The existence of such a principle in the blood of the scrofulous, forms, however, no part of our pathological creed. Rev.

rarely elsewhere."* Young persons, who are brought to England from tropical climates, almost invariably become scrofulous and consumptive.

Thirdly. The proximate cause of scrofula solely depends on cutaneous absorption.—The absorbent glands of the neck are constantly the seat of the primary symptoms of the disease; a phænomenon only explicable by ascribing it to the action of the cutaneous absorbing vessels. This exclusive proneness to the invasion of scrofula, in the cervical glands, is the consequence of their exposure to atmospheric inclemencies and vicissitudes. The delicate organization of the epidermis of the face, and of the membrane lining the lips and buccal cavity, moreover, singularly favours absorption. Scrofula evinces a "preference for certain districts." Such as are "very elevated, where the air is thin and sharp; or such as are low and marshy, where it is thick and foggy," are particularly obnoxious to its ravages. Now, hydrogen gas abounds in these situations; for being, on one hand, the lightest of the gases, it must necessarily occupy the higher strata of the atmosphere, and being, on the other, the production of marshy soils and stagnant waters, it cannot but form a considerable portion of the lower, in such situations. Hence the absorption of hydrogen gas, or of atmospheric air, deriving tenuity from its large admixture, is the proximate cause of scrofula! This is truly one of the Doctor's finest — one of his first-rate soap-bubbles. But we like it not

* We cannot but admire the happy facility with which, when it suits his convenience, Dr. Henning revokes an opinion formally delivered. In the first chapter, he declares that bronchocele is *not* of a scrofulous nature: here he admits that it *is*, without assigning any reason but his own "mature consideration." Of the accuracy of this latter admission, we have no doubt. But Dr. Henning falls into an egregious error in asserting that the disease is "endemial to very elevated situations, and is rarely met with in others." The celebrated Foderé, in his little work, *Du Goitre et du Cretinisme*, expressly states, that the affection occurs exclusively in the *vallies* of the Alps, and *is cured by removal to a more elevated region of these mountains.* In confirmation of the efficacy of this mean, he cites his own case. A native of one of the Alpine districts, he was, in his earlier years, affected with bronchocele. Our own experience completely coincides with this unquestionable testimony. We practice in a district where, from the lowness and humidity of the situation, bronchocele is a common affection, and scrofula commits dreadful ravages. On the more elevated country around us, the disease is comparatively rare; and we find a removal from low to higher ground, one of the most effectual means of arresting its deadly progress. Unfortunate facts these for the credit of the Doctor's accuracy of observation, and his hydrogen theory of scrofula! Rev.

the better for being filled with hydrogen, which will only accelerate its explosion on coming in contact with the common atmosphere. But let us proceed.

Fourthly. The superficial absorbent glands alone are susceptible of the original action of scrofula: if other organs become affected by it, such affection is consequential. No other region but the neck is subject to the attack of scrofula. Any disease, originally invading the structure of the inguinal, axillary, or mesenteric glands, is not scrofula; and why? because, according to Dr. Henning's *theory*, those, superficially seated, are alone capable of being primarily affected by it. But how happens it, that swelling of the cervical absorbent glands does not invariably precede a scrofulous affection of the system? Oh, replies the ingenious all-explaining Dr. Henning, " it is highly *probable* they are (first affected); but it neither follows of necessity, that they shall suppurate, burst, and leave stigmata, nor that they shall remain in an enlarged and indurated state. It is altogether *possible*, that scrofulous virus (which we verily believe has no existence but in the Doctor's imagination), after first exciting tumefaction of the absorbent glands, is insufficient to maintain permanent increased action in them; this will, therefore, after a time, subside spontaneously, and permit it (the virus) to pass." And this frequently results from the ill-directed efforts of art to subdue the excitement of an enlarged absorbent gland. The anatomical characters of a morbid mesenteric gland, and of one really suffering from scrofula, are completely different.

Fifthly, and lastly. A close analogy exists between scrofula and lues venerea: for the fomes of both is communicated to the body by the superficial absorbent vessels; the progress of both is opposed by the process of glandular tumefaction, and the consequent diseases of the system are most materially " influenced * by the same medicine,"— mercury, of course. The inference is, that it is of importance, in both these complaints, to cure the enlargements of the absorbent glands by " encouraging suppuration;" and it is probable that, " by these means, the constitution escapes contamination."

To these propositions, and the assertions on which they are founded, our answer will be more brief than we at first

* The prudence of Dr. Henning, in the employment of the saving word *influenced*, is highly to be applauded. On this point we shall have occasion to animadvert farther bye and bye. Rev.

designed. Our patience is well-nigh exhausted in poring over such a farrago of crude and monstrous absurdities. In this reply, we shall confine ourselves to facts, and those few and simple. Of defeating, by solid reasoning, such a light-heeled and gaseous antagonist as Dr. Henning, we utterly despair. Equally idle would be the attempt, as attacking shadows with a crab-stick. But we hope to convince our readers, if one there be among them so short-sighted as yet to remain unconvinced, that the arguments of the Doctor are futile and unsubstantial as the aërial hypothesis which they are employed to sanction and defend.

In the first place, then, it is certain that few individuals, comparatively, become the victims of scrofula, whose progenitors have not, at some period, suffered more or less from the ravages of the malady. A predisposition to become affected by certain diseases, on the application of exciting causes, does certainly and almost universally exist in the human race. In some instances it is more strongly marked than in others. We have every reason to *believe* it, like peculiarities of physical character, transmitted from the parent to the progeny. But predisposition is inert, and, of itself, inefficient to produce disease. It requires for this purpose the application of an exciting cause. Thus the son of a gouty father may preserve himself from the malady by a life of temperance and activity. Again, the exciting cause may be so direct or continued in its application, or so powerful in effect, as to produce disease where no predisposition to it previously existed. The infliction of severe mechanical violence on the thoracic parietes, for instance, may induce pulmonary hæmorrhage and phthisis; or the ingestion of an active mineral poison, chronic visceral disease, in persons not originally predisposed to the attack of a pulmonic or intestinal lesion. On like principle too, the continued influence of a damp and variable atmosphere may induce scrofula in a native of the equatorial regions. However "absurd" Dr. Henning may affect to consider these doctrines, they are founded on accurate observation; and the fact with which he, at page 59, attempts to refute them, constitutes, if we mistake not, a strong evidence of their correctness.

2. Peculiarity of climate, that is, a damp and variable condition of the atmosphere, such as commonly prevails in low situations, on the shores of considerable lakes or rivers, or in the deep vallies of the Alps, may be regarded, we verily believe, as one of the exciting * causes of scrofula. Unwholesome and indigestible aliment, corporeal inactivity, and every other cause which operates by impairing the functions of the skin or chylo-poietic organs, are more or less adequate to its production. We have seen decidedly scrofulous swellings of the cervical glands permanently yield, *without suppuration* and *without change of residence*, to the conjoint operation of diet, exercise, and medicine. How could this be, if scrofula, according to the Doctor's hypothesis, were exclusively dependent on atmospheric peculiarities? Could an effect be removed, while the operation of its cause existed in full activity and force? Surely not, according to our vulgar notions of effect and cause.

3. That the cervical absorbent glands are exclusively prone to the primary attacks of scrofula, and that hydrogen gas, or atmospheric air largely mixed with it, would, if absorbed, exert a noxious influence on the glandular system, and thus generate a virus, or specific poison, capable of contaminating the whole fabric, is mere gratuitous bare-faced assertion, utterly unsupported by legitimate induction from fact, or even the less conclusive evidence of analogy; and yet upon this sandy foundation does the tottering edifice of Dr. Henning's hypothesis solely repose. Again, it is more than questionable whether, under common circumstances, aëriform or other fluids, are taken up by the cutaneous absorbents. Many facts, which have fallen within our reading and observation, seem strongly hostile to such a supposition. We have, moreover, shewn, that in elevated situations, where, according to the Doctor's crude notions of aërology, hydrogen gas chiefly abounds,† scrofula does, by no means,

---

* If Dr. Henning's theory of the production of scrofula were correct, how happens it that all, who are exposed to the influence of a morbific atmosphere, do not invariably suffer from its ravages? Some escape, he argues, from not being exposed under the necessary circumstances; (what are those circumstances?) some from superior strength; while others are rendered insusceptible of its action from *custom*, which has a wonderful faculty of disarming noxious effluvia of their power. Now, a period of *initiation* to the influence of these noxious effluvia, we imagine, there must necessarily be; and what, it may be inquired, becomes of the poor cervical glands for which hydrogen and scrofula evince such an unfortunate "preference," *previously* to their becoming duly seasoned? REV.

† It must be a hill of no ordinary elevation, the atmosphere of which, from its decreased density and weight, would allow any *permanent* accumulation of hydrogen gas.

generally prevail: and it is self-evident, that in the low districts, which the unsightly hag honours with her "predilection," this supposed friend and colleague, hydrogen, cannot, from his specific lightness, muster his aërial forces in sufficient strength to become formidable. The cheeks and lips can merely sustain an occasional brush from their gaseous enemy, in his rapid march to the more elevated regions of the atmosphere

4. Is there, among our readers, a physician or surgeon of five years' standing in practice, who has not repeatedly seen scrofula developed in its darkest and most intractable form, and run its fatal course without the slightest previous, or even consequent affection of the cervical absorbent glands? Is there one, who has not repeatedly seen swellings of the neck arise long after the invasion of other parts, the mesentery, the arm-pit, or the groin, by scrofulous action? Is there any one, except Dr. Henning, (and we much doubt whether a mind poisoned, like his, by the trash of hypothesis, could stoop to the coarse substantial fare of the dissecting table) who can descry any real difference of structure in a scrofulous gland, whether taken from the groin, the abdomen, the axilla, or the neck? And shall we, rejecting the light of accumulated observation and correct inference, be made to believe that affections of these regions, precisely resembling each other in their commencement, progress, and termination, in the constitutional phænomena which accompany them, in anatomical character, and in the treatment which they require,—are of a nature and origin essentially different; and this on the bare assertion of a man, whose name and existence were first announced to us by the title-page of his book, and whose sole object is evidently the fabrication and support of an hypothesis, which, by its novelty, may attract the public eye to its author, ere the slippery ground on which it is erected gives way, and the rotten materials, of which it is composed, return to their native mud? As to Dr. Henning's conjecture, that the poison of scrofula may contaminate the system by the medium of the cervical absorbent glands, without exciting permanent disturbance in, or leaving any trace of its passage through, them, it is such mere finesse,—such a paltry subterfuge, and flagrant deviation from the avowed principles with which he set out, that we only condescend to notice it with a view of exposing his unblushing contradictions and inconsistencies. Does he not expressly assert, at page 55, when combating the doctrine of morbid hereditary predisposition, that,

"unless it (scrofula) be evinced by its appropriate symptoms, which are swellings of the superficial glands, there is no evidence of it. If these be present, they must be manifest. If, then, there be no local affections, for these are not only the diagnostic symptoms of scrofula, but constitute its very essence, it is not present; and to contend that it is, is *but to support a contradiction.*" And yet this accurate and consistent gentleman admits, although not without some hesitation, that the absorbent glands of the neck are not always affected before the system exhibits symptoms of scrofula; nay, he is even unguarded enough to cite from another author, in corroboration of his opinions respecting the influence of climate in the production of the malady, a passage which is decidedly at variance with his doctrine, that its original attack is *invariably* made through the medium of the cervical absorbent glands.* With regard to the possibility or probability of these glands, although at the actual period of observation sound, having been originally affected, we have only to assail the Doctor with his own weapons, and retort upon him the "saying of the schoolmen, *de non apparentibus, et de non existentibus, eadem est ratio.*"

5. Lastly. It would be idle to waste time in elaborate attempts to disprove the supposed analogy between syphilis and scrofula. The thing carries absurdity in its face. It is one of those mis-shapen monstrous productions which could only be engendered in the hot-house of a distempered brain, and must perish in the first stream of external air that blows upon it. The premises upon which this doctrine rests are unsound; the conclusions drawn from them utterly delusive. Both must fall together.—*Syphilis* is spread by the direct application of a morbid poison to

* Mr. Pearson, speaking of the African children, who, on removal to this country, almost invariably die "scrofulous," observes: "*Sometimes* the consumption was preceded by swelling and suppuration of the glands, enlargements of the bones, &c.; but in the females, the consumption was often the *primary* complaint." To this "decisive testimony" the Reviewer begs leave to add his own. Within the last two years, he has examined, with his own hand, the bodies of six scrofulous subjects. In five of these, the cervical absorbent glands had never been known to exhibit the slightest tumefaction. The sixth was affected with swellings of the neck, but these were only first developed towards the close of the malady; and, on dissection, both the mesenteric and cervical glands exhibited precisely the same anatomical characters, those of genuine scrofula. In one of the most intractable scrofulous cases that has recently fallen under his observation, the *foot* was *first* affected with abscess; then the groin, and *last of all, the cervical glands.*

the human organs; in fact, by inoculation:—In *scrofula*, the existence of no such poison has been demonstrated; and hence, the term scrofulous virus is perfectly inadmissible.—*Syphilis,* like cow-pox, is a constitutional malady of local origin; exclusively propagated by contagion, and unrestricted in operation:—*Scrofula* consists, like gout, in some local affection, invariably preceded or accompanied by the phænomena of constitutional derangement; and is endemic, or peculiar to certain climates and situations. —*Syphilis* never spontaneously disappears; and mercury, exhibited so as to affect the system, is its specific remedy; —*Scrofula,* after committing dreadful ravages on the system, has frequently been known to wear itself out; and it is signally aggravated by such large administration of mercurials as will excite the salivary organs, and is commonly requisite for the cure of the venereal malady.* And, in the last place, we positively assert, from experience, that the suppuration of a cervical absorbent gland, when first attacked by scrofula, will not, of itself, afford security to the constitution by arresting the progress of the supposed poison, or in any other way. Much more reasoning, equally conclusive, might be here advanced; but we have already squandered too much time on this fantastical thing of an hypothesis, and must now proceed to take a cursory view of Dr. Henning's "practice in scrofula."

*Part the Second,* devoted to this subject, includes *five chapters.* The four first, in which Dr. Henning's "new and much improved" plan of therapeutics is explained, we shall take together, and present, under the heads of surgical and medical treatment, a rapid outline of their contents. The fifth, we deem entitled to separate cognizance.

*Surgical Treatment of Scrofula.*—The reader, who has duly attended to the developement of Dr. Henning's theory, will descry at a glance the practical conclusions to which it leads. The tumefaction of the cervical glands in scrofula, like that of the inguinal in syphilis, is a salutary process, instituted by nature to arrest the progress of the poison, and prevent contamination of the system.† These

---

* The complication of scrofula with syphilis is greatly dreaded by practitioners, as the presence of the former renders it difficult or impracticable to administer with impunity as much mercury as is absolutely requisite for the cure of the latter. Rev.

† The late Mr. Crowther recommended the dispersion of the contents of scrofulous abscesses by continued blistering; and asserted, that no inefforts must not be frustrated by the officious interference of art. Leeches, cold applications, all topical and general repellents, must be utterly discarded. A mild adhesive plaster, and subsequently cataplasms are to be employed; and when the process of suppuration is sufficiently advanced, the contents of the abscess are to be evacuated by a lancet, at once, if the collection be not large; if otherwise, by repeated puncture, at proper intervals,* and the access of external air prevented by careful closure of the orifice. In fact, the "*new*" mode of operating is precisely that which Mr. Abernethy has long since adopted in the treatment of lumbar abscess. Thus, Dr. Henning pretends, the system will be preserved from contamination by the scrofulous virus: and the operative process by which these wonders are atchieved, he affects to consider as, at least in its application to scrofulous swellings, exclusively his own. But this claim of originality stands, like the Doctor's whole hypothesis, on a somewhat insecure footing; since, from our earliest acquaintance with the London schools of surgery, Mr. Abernethy has been accustomed to recommend annually, in his lectures, this identical mode of evacuating the pus of scrofulous abscesses, with a view of preventing the deformity consequent on its spontaneous discharge by absorption of the integuments: and we, from this recommendation, and *on this principle*, have repeatedly practised it within the last ten years.

Before quitting this section, we beg leave to address a few simple observations to the Doctor, on his pathology

---

jurious consequence was ever seen by him to result from such practice. Now this fact, if correctly stated, as doubtless it is, must utterly explode the Doctor's hypothesis. The latter, therefore, first disingenuously attempts to throw discredit upon it; and, subsequently, to fritter away its obnoxious evidence by an acknowledgment that the irritation, kept up by the raw surface, has "a very powerful tendency to divert from the constitution, consequences which, without it, could hardly fail to result." But, unless the Doctor can prove the evacuation, thereby, of his pretended morbid poison, this pitiful finesse will avail him nothing. Rev.

* If Dr. Henning's hypothesis respecting the existence of scrofulous virus, were correct, *partial* evacuation of the contents of the abscess might be very justly denounced as rash and dangerous practice. Removal of a *part* of the load from the absorbents, in any unnatural accumulation of fluid, is a very effectual mean of exciting these vessels to take up the remainder. Thus the remaining fluid of ascites and hydrocele has sometimes disappeared in a few hours after partial evacuation by the trocar. The stimulus of the operation, and even the excitement necessarily attendant on the process of adhesive inflammation, perhaps contribute, in some degree, to the production of this effect. Rev.

and surgical treatment of bronchocele. He acknowledges this affection to be of a scrofulous nature; and he recommends that, in the event of suppuration being menaced, leeches, and other anti-inflammatory remedies (for these, we imagine, are implied by the &c.) should be put in requisition to avert it. Now the grand principle of his theory, it must be recollected, is, that the cervical absorbent glands *invariably* constitute the primary seat of scrofula; and that, through the medium of them *alone*, the system itself, or any of the various organs composing it, can become affected by this malady. But the thyroid is not an absorbent gland, nor placed in the direct route of the cervical lymphatics; and again, surely no man in his senses will be hardy enough to maintain that bronchocele is commonly preceded, or accompanied, by tumefaction or other morbid appearance of the superficial conglobate glands in the vicinity. How then does Dr. Henning's admission of the scrofulous nature of bronchocele correspond with the fundamental principles of his theory? And, according to these same principles, would not the plan of repercussion "frustrate the obvious purpose of the constitution," and endanger, or indeed inevitably decide, its contamination by absorption of the scrofulous virus? These strange incongruities of doctrine and practice, it imperiously behoves our unfortunate theorist to reconcile and explain. Neglect or failure to do this must bring down upon himself a lasting imputation of the most lamentable blindness and inconsistency, and doom to irrevocable destruction his ricketty hypothesis.

*Medical Treatment of Scrofula.*—During the maturation of a scrofulous abscess, neither mercurials, active purgatives, or other medicines which may disturb the process, or promote absorption, are to be administered. Such remedies should be reserved till after the period of its evacuation; when they may be usefully employed to obviate, or counteract, the influence of absorbed virus on the system, and remove the deformity which might otherwise result from suppuration of the gland. The medicinal articles by which the constitutional affections, arising from absorption of scrofulous virus, or "secondary symptoms of the disease," may be most successfully combated, are arranged in two classes, the *tonic* and *deobstruent*. To the first class, belong the *cinchona*, which is only correctly indicated when debility is present, or when the swelling has suppurated, but *before* its contents are evacuated, to counteract the debilitating tendency of the scrofulous virus. *Iron,* only to be exhibited where there is weakness and want of tone, and improper if febrile symptoms exist. *Port wine,* most advantageously given between meals. *Vegetable bitters,* admissible where the heat of the surface is too high to allow the use of more active tonics, to which they may be made preparatory. *Mineral acids,* of which the nitric and sulphuric are to be preferred, as being tonic without stimulant effect, and hence indicated where other tonics are inapplicable; and, lastly, the *cold bath,* rather to be considered as a preventive than a curative remedy. The deobstruent class includes, *quicksilver,** *eccoprotics, burnt sponge,* and *mezereon.* Dr. Henning's observations on these substances display no particular novelty or interest. The sponge, combined with cinchona or aperients, is evidently a favourite preparation. *Sarsaparilla,* he thinks, " is remarkably serviceable in those cases which appear to partake of the nature of *scrofula and lues.*" His testimony respecting the efficacy of *conium* and the *muriates of barytes and lime* is unfavourable; and respecting that of the *warm bath,* indecisive. On the subject of the pure alkalis, potash and soda, Dr. Henning is utterly silent. Truly unfortunate that he should have raked his physic-box of all the rubbish, and left the pearl behind! *Purulent drains* he regards as of singular utility in scrofula; and supposes that the fomes, absorbed into the system, may be deposited in them, and thus "find an exit instead of falling on the lungs or other important parts."

---

* Of all the preparations of quicksilver, Dr. Henning prefers the muriate, submuriate, and red and grey oxyds—" the forms which chemistry gives it." He objects to those in which " its particles are separated by mechanical means, as in the pilula hydrargyri, and hydrargyrus cum cretâ." But can the doctor be ignorant that the quicksilver of these despised formulæ is absolutely in a state of oxyd, little differing from, and quite as much a chemical preparation, as the grey oxyd separately obtained by a more elaborate process? Alas! the doctor is no chemist. Afterwards, in an attempt to explain the frequent supervention of scrofula to lues, he imputes the excitement of the former, not to any direct influence of the latter, but " to the operation of the quicksilver (employed for its cure) on the surface of the body." Another unlucky blow this for the supposed analogy between scrofula and syphilis. No sooner out of one difficulty, than chin deep in another! He farther observes, that should this supposition be hereafter confirmed, " it will afford additional probability to my *conjecture,* that scrofula depends on an increased, instead of an impaired action of the superficial absorbents." How mightily amusing to see the fundamental doctrine of the hypothesis which has been repeatedly advanced with all the boldness and dignity of a demonstrated truth, thus dwindle at once into mere, flimsy, probable, conjecture!

Sorry pathology this indeed! and yet more miserable the practice which it is manufactured, or rather brought from the shelf where it has peacefully slumbered during a greater part of the last century, to explain. Issues and all other permanent drains from the system, in fact, are indiscriminately denounced as pernicious in scrofula, by a man who knows more of the disease, has had more extensive experience and greater success in the treatment of it, and has written a better practical work upon it than any other physician or surgeon in the three kingdoms.* And the general accuracy of his opinions, and value of his plan, many a successful experiment has taught us to appreciate and respect. Yet we think that he goes too far in rejecting, in all cases and under all circumstances, the employment of issues. In one instance, at least, we have seen decided mischief result from this indiscriminate proscription.

We now come to the *last Chapter* or *Appendix;* which is said to contain "proofs and illustrations of the doctrine advanced in the preceding part of the work, and cases exemplifying the practice there recommended." These cases are seventeen in number. Three are borrowed from other writers. Of the remaining fourteen, six are scrofulous; and eight consist of syphilitic, cancerous, and different anomalous affections. It is remarkable that we find not among them one case, in which the cure of scrofula was attempted by the mere evacuation of the abscess, *unassisted by constitutional remedy*; and this, let it be remembered, is the point principally contested—the position by the establishment or loss of which, the fate of the whole hypothesis must be eventually decided. The cases, in truth, are generally of such a nature that they would serve equally well to prove any other doctrine, or illustrate any other practice, which the vanity or interest of an author might tempt him to foist upon the public credulity. Bad must be the trim, and desperate the situation, of the main force which rests for its support upon such a motley and undisciplined body of reserve as this!

Nothing forms a more prominent feature in the volume upon which we have been commenting, or has more strongly excited our loathing and indignation during its

---

* See *Observations on the use of caustic alkali in scrofula and other chronic diseases*, by Joseph Brandish; a production which reflects honour upon its liberal, intelligent, and unassuming author.—Rev.

perusal, than the unphilosophical method of investigation and argument which Dr. Henning has uniformly adopted. Assuming as the principle of his hypothesis a point wholly conjectural and unsound, he proceeds to draw his inferences from it; and whenever the accuracy of any one becomes questionable, he makes it to repose upon some other equally fallacious, or reverts, in a tone of assumed infallibility, to his original premises. Of this, numerous instances might be adduced. Now some philosophers have contended, that every deviation from right reason may be regarded as a leaning, or actual approach towards insanity: and it is still less to be doubted that arguing from false data, and acting upon the suggestions to which they give rise, constitute one of the strongest characters of mental hallucination. Upon this principle, the evil habit of *circular* reasoning will, perhaps, be most effectually combated by a few daily whirls in the *circular* swing. We shall conclude by telling Dr. Henning, nearly in his own language, that his account of the cause, nature, and seat of scrofula is neither "rational nor philosophical," scarcely "intelligible; in fine, that his whole hypothesis is visionary and foundationless."

Yet are we willing, ere the Doctor be dismissed, to temper the severities of our wholesome reproof with a spice of admonition and encouragement, and, by mingling a little ginger in our senna potion, prevent undue griping. He possesses talent, learning, assiduity. The gleam of an observant and sagacious spirit occasionally breaks through the obscurity of his errors. Let him no longer abuse these precious gifts. Let him contemplate Nature, not through the dark and distorting medium of imagination, but calmly, philosophically, in her own pure and unclouded mirror; and delineate with truth, and arrange and digest by the light of science, the observations which he has made. Thus will he acquire a lasting claim to the respect and gratitude of those around him, and avert from his declining years the curse which conscience, brooding over the recollection of squandered talent and lost opportunity, must otherwise, sooner or later, inflict. And we, he may rest assured, shall be amongst the first to pour "oil and wine" into the wounds which he has rashly incurred from the scourge of criticism; to blazon his reformation; and unite our feeble suffrage with the grateful voice of public testimony, in his applause.

